# Lifetime reproductive success is maximized with optimal major histocompatibility complex diversity

**DOI:** 10.1098/rspb.2008.1466

**Published:** 2008-11-25

**Authors:** Martin Kalbe, Christophe Eizaguirre, Ilka Dankert, Thorsten B.H. Reusch, Ralf D. Sommerfeld, K. Mathias Wegner, Manfred Milinski

**Affiliations:** 1 Department of Evolutionary Ecology, Max Planck Institute for Evolutionary BiologyAugust-Thienemann-Strasse 2, 24306 Ploen, Germany; 2 Institute of Evolution and Biodiversity, Plant Evolutionary Ecology, University of MuensterHuefferstrasse 1, 48149 Muenster, Germany; 3 Institute of Integrative Biology, Experimental Ecology, ETH-ZentrumUniversitaetstrasse 16, CHN J12.1, 8092 Zurich, Switzerland

**Keywords:** lifetime reproductive success, major histocompatibility complex, stickleback, parasites, mating decision

## Abstract

Individual diversity at the major histocompatibility complex (MHC) is predicted to be optimal at intermediate rather than at maximal levels. We showed previously in sticklebacks that an intermediate MHC diversity is predominant in natural populations and provides maximal resistance in experimental multiple parasite infections in the laboratory. However, what counts ultimately is the lifetime reproductive success (LRS). Here, we measured LRS of six laboratory-bred sib-groups—to minimize the influence of non-MHC genes—three-spined sticklebacks (*Gasterosteus aculeatus*) during their entire breeding period, each in a seminatural enclosure in the lake of their parents, where they were exposed to the natural spectrum of parasites. We collected developing clutches at regular intervals and determined parenthood for a representative number of eggs (2279 in total) per clutch with 18 microsatellites. Both males and females with an intermediate MHC class II*B* variant number had the highest LRS. The mechanistic link of MHC diversity and LRS differed between the sexes: in females, we found evidence for a trade-off between number of eggs and immunocompentence, whereas in males this correlation was concealed by different timing strategies of reproduction.

## 1. Introduction

Genes of the major histocompatibility complex (MHC) are the most polymorphic genes and play a fundamental role in the adaptive immunity of all jawed vertebrates. They encode cell-surface proteins, which present either self-peptides or peptides derived from phagocytosed pathogens to T lymphocytes, a prerequisite for production of pathogen-specific antibodies and the development of an immunological memory (Janeway *et al.* 2005). The enormous allelic diversity found in natural populations is generally regarded as a consequence of parasite-mediated balancing selection (Clarke & Kirby 1966; Takahata & Nei 1990; Apanius *et al.* 1997; Edwards & Hedrick 1998; Jordan & Bruford 1998; Penn & Potts 1999; Penn *et al.* 2002; Bernatchez & Landry 2003; Mays & Hill 2004; Milinski 2006). Several reports have shown correlations between certain MHC genotypes and occurrence or severity of specific diseases and parasite infections (Briles *et al.* 1983; Hill *et al.* 1991; Godot *et al.* 2000; Langefors *et al.* 2001; Grimholt *et al.* 2003; Bonneaud *et al.* 2005, 2006; Harf & Sommer 2005). This implies that individual MHC diversity should be maximized in order to achieve resistance against as many different pathogens as possible. However, although some studies show that MHC heterozygotes are more resistant than homozygotes (Doherty & Zinkernagel 1975; McClelland *et al.* 2003), the overall evidence remains ambiguous especially for species harbouring recently duplicated MHC loci (Penn 2002; Penn *et al.* 2002; Pitcher & Neff 2006). A recent study showed that mice that were heterozygous at all MHC loci were even less resistant than mice that were homozygous at all loci when challenged with different strains of salmonella (Ilmonen *et al.* 2007). Therefore, it seems to be disadvantageous to have too many different MHC alleles. Indeed, each time a distinct MHC molecule is added to the individual MHC repertoire, all T-cell clones that can recognize self-peptides bound to that molecule must be removed in order to maintain self-tolerance. This process of negative T-cell selection can prevent an efficient adaptive immune response if an individual has too many different MHC alleles (Lawlor *et al.* 1990). Thus, theoretical models predict that maximal pathogen resistance is achieved by an intermediate, i.e. optimal, rather than a maximal number of different MHC variants (Nowak *et al.* 1992; De Boer & Perelson 1993; Borghans *et al.* 2003; Milinski 2006; Woelfing *et al.* 2009). The most recent mathematical model (Woelfing *et al.* 2009), which is based on novel findings on T-cell selection, can predict the natural range of intra-individual MHC diversity. This prediction is in line with empirical studies, showing the lowest effect of parasite infections, a fitness trait, in fish (Wegner *et al.* 2003*a*,*b*, 2008), and in reptiles (Madsen & Ujvari 2006) with intermediate MHC diversity. Such a beneficial amount of individual MHC diversity may not only be maintained by parasite-mediated selection, but also be amplified by MHC-based mate choice. For instance, female sticklebacks prefer males with a complementary number of MHC class II variants to their own set of variants, resulting on average in intermediate immunogenetic diversity in their offspring (Reusch *et al.* 2001; Aeschlimann *et al.* 2003; Milinski *et al.* 2005). Recently, Forsberg *et al.* (2007) found an evidence for a similar mating preference of female brown trout (*Salmo trutta*) for males with intermediate MHC dissimilarity. Thus, fitness should ultimately, i.e. during an individual's lifetime, be maximized when MHC allele diversity is intermediate.

In this study, we tested the effects of individual MHC class II diversity on lifetime fitness by measuring the lifetime reproductive success (LRS) of three-spined sticklebacks (*Gasterosteus aculeatus*) under natural conditions. Sticklebacks offer an exceptional system because, in populations of our geographical latitude, they usually have only one reproductive period in their life (Wootton 1976). Moreover, life-history traits involved in reproduction have been studied extensively, such as breeding coloration that has been shown to be a good predictor of parasitation and body condition (Milinski & Bakker 1990; Frischknecht 1993; Folstad *et al.* 1994; Kraak *et al.* 1999; Barber *et al.* 2000). In a field enclosure system, we analysed mating combinations and individual reproductive output of laboratory-bred sticklebacks. Within each of six enclosures, fish (eight males and eight females) were full siblings, to reduce the influence of non-MHC genes (genetic background), and fish differed almost only with respect to their MHC genotype. Experimental fish had been individually challenged twice with three common sympatric macroparasites from the lake of their parents to simulate a natural life history comparable to their free-living conspecifics. In the enclosures, we collected all developing clutches of eggs until the end of the reproductive season and allocated the most likely parents to each clutch. Therefore, using genetic methods we could estimate the LRS of each individual fish. Immunogenetic optimality should ultimately contribute to LRS. Therefore, we expect that the fish with an intermediate MHC class II*B* diversity have the highest reproductive success. This would explain why individuals with an intermediate MHC diversity predominate natural populations (Reusch *et al.* 2001).

## 2. Material and methods

### (a) Experimental fish

Three-spined sticklebacks caught from a natural population from the lake Großer Plöner See were used for breeding as described elsewhere (Kalbe & Kurtz 2006). Several sibships per breeding pair were raised until the age of three months when offspring from the same pair were combined and transferred to 190 l tanks in densities of 100–250 fish. Fourteen randomly chosen individuals and both parents of each sibship were analysed for their MHC class II*B* genotype (see below). Six sibships with segregating numbers of MHC class II*B* alleles/variants were selected, as in Wegner *et al.* (2003*a*). In such families, with a similar genetic background, MHC genotypes with different numbers of sequence variants were present: intermediate diversity (approx. six sequence variants); a low number (less than five sequence variants); and a high (more than seven sequence variants).

### (b) Experimental parasite infections and time schedule

**Figure 1:**
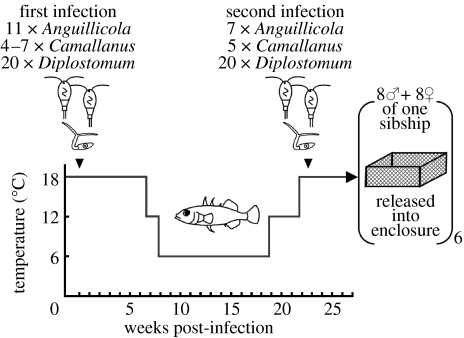
Schematic of the experimental infection schedule and temperature (grey line) regime in the laboratory, prior to release of the sticklebacks into the enclosure.

From each sibship, 30 randomly selected fish were each experimentally exposed twice to a combination of three of the most prevalent macroparasite species originating from their parents' habitat: the nematodes, *Anguillicola crassus* and *Camallanus lacustris*, as well as a digenean trematode, the eye fluke *Diplostomum pseudospathaceum*. All parasites originated from the Großer Plöner See or contiguous neighbouring lakes and, therefore, are regarded as sympatric to the stickleback population examined here. Infection of fish was performed as described elsewhere (Kalbe & Kurtz 2006; Krobbach *et al.* 2007). Fish were exposed to the combination of all three parasites in December 2004, and again in May 2005. Between treatments, to mimic natural life history including parasite exposure, sticklebacks were brought stepwise to laboratory winter conditions (6°C, 10 h light d^−1^) before re-experiencing summer conditions (18°C, 16 h light d^−1^; see figure 1 for infection dosages and schedule).

### (c) Enclosures

The outdoor experiment was conducted in the lake Großer Plöner See, in northern Germany (54° 9′21.16′ N, 10°25′50.14′ E) during the summer 2005. The enclosure system consisted of six stainless steel mesh cages (3 ×3 m, total height 1, 0.4–0.6 m above water surface) installed on the lake ground in a row close to the shoreline, located in the natural breeding area, i.e. where the parents of the experimental fish had been in the previous year. The 5 mm meshes allowed only small particles and most invertebrates (food items and intermediate hosts of various parasite species) to pass through. A coarse meshed net protected the fish from bird predation. Prior to release into the enclosures, we weighed (±0.1 mg), measured (±1 mm) the fish and counted the *Diplostomum* metacercariae in each eye lens under a dissection microscope. Finally, a 2–3 mm piece of the first dorsal spine was cut for genotyping at the end of the experiment. Each of the six enclosures was stocked with eight males and eight females, one family per cage, on 16 June 2005.

### (d) Egg collection

Every week but one, because the weather did not allow it, all stickleback nests were detected in each enclosure by careful observation. All egg batches were removed and the nests were carefully replaced in their original location. Egg batches were brought to the laboratory; clutches were separated on the basis of different developmental stages. If necessary, the individual egg clutches were incubated in aerated well water (with 0.04 ppm malachite green) at 18°C until dark eye spots and the neural tube developed, to ensure a sufficient amount of DNA for further analysis (see §2f).

### (e) Recapture and examination of sticklebacks

During the last egg collection on 3 August, when nests no longer contained fresh clutches, all surviving fish were caught. Immediately following capture, a picture of each male's red throat was taken within a dark box using a digital camera (Olympus E20p) with a 36 mm macro lens. For the camera parameters and the handling of fish, see Jäger *et al.* (2007). Intensity analysis of the red coloration was performed with IP Lab v. 3.6.2 for Mac OS v. 9.2.2 (Scanalytics, Inc.) delimitating a defined area of the red throat. Jäger *et al.* (2007) showed that method to be highly repeatable. Surviving fish were measured, weighed and dissected within 3 days after capture, including screening for macroparasites and weighing of organs (gonads, kidney, liver and spleen). General body condition of the fish was calculated according to Bolger & Connolly (1989).

All external and internal macroparasites and ciliates were determined to the lowest taxonomic level possible. In order to quantify the total parasite load of each fish, an individual parasite index was calculated (Kalbe *et al.* 2002). This allowed different combinations of rare and frequently occurring parasites to be summarized for each stickleback and their total parasite burden to be compared quantitatively.

### (f) Major histocompatibility complex and microsatellite typing

DNA extractions from dorsal spines of fish before release into the enclosures and after recapturing, as well as from the developing eggs, were conducted using DNA Tissue kit (Invitek, Germany) following the manufacturer's protocol. All fish and a representative subsample of the collected fry (see below) were typed for 18 microsatellites (see the electronic supplementary material) combined in five different PCR protocols (Largiadèr *et al.* 1999; Peichel *et al.* 2001). This number of microsatellites was needed in order to guarantee a high parenthood resolution even within sibships. At the end of the experiment, survivors were retyped for identification. The MHC class II*B* diversity was determined using capillary electrophoresis single-strand conformation polymorphism of the amplified exon 2 of the MHC class II*B* chain as described in Binz *et al.* (2001) (see Reusch & Langefors 2005 for an estimation of the number loci).

### (g) Parenthood analysis and heterozygosity

From each egg clutch, 16 eggs were randomly picked for parentage analysis. Paternity assignments were performed with the software PAPA for every egg (Duchesne *et al.* 2002). Accordingly, each egg was assigned to one of the following male categories: nest owner, sneaker and stolen. Males that fertilized the majority of the eggs in a nest were categorized as nest owners and these eggs were assumed to originate from females that have chosen this male. Eggs were assigned as being fertilized by a sneaker, if in one nest the same female had eggs fertilized by the nest owner and another male. Eggs were assigned as being stolen from other nests if the nest owner did not fertilize them and no further eggs of the same female were found in the nest. Moreover, we verified that the combination existed in other nests to exclude wrong affiliations from the software. We also calculated an heterozygosity index for each individual parent (Coulson *et al.* 1998).

### (h) Data analysis

All statistical analyses were conducted in JMP v. 5.0.1 (SAS Institute). To start, a large multiple correlation was performed and occurring collinearities were corrected by taking the residuals of the regressions. Stepwise model selections was performed based on the AIC criterion (Sakamoto *et al.* 1986).

LRS was calculated for both surviving and total number of fish since some dead fish had a certain fertilization success.

#### (i) Lifetime reproductive success

We aimed at estimating the LRS of each fish. First, we performed an ANCOVA on the total number of eggs assigned to each parent, with enclosures, sex, parasite load, initial and final body condition (expressed as residuals of the regression with initial body condition) as the dependent variables. Second, we correlated the residuals of the previous model for LRS with MHC class II*B* diversity with both linear and quadratic terms.

With our design, we had to test the correlation between MHC genotypes and LRS within sibships. We used Wegner *et al.*'s (2003*b*) method extended from Aeschlimann *et al.* (2003) and (Milinski 2003) to calculate the reproductive success dependent on the number of individual MHC variants using each sibship as a statistical unit. Briefly, we averaged the number of eggs within each sibship over each MHC genotype and calculated all the possible slopes between these points. For a first ‘purist’ test, we used the average number of eggs and the average slope derived as described and used this as one pair of data points from each sibship (see §3 for detailed description of analysis and the electronic supplementary material for a fictitious example explaining this method). Further tests took individual fish and thus more information into account. Because we had clear predictions with intermediate genotypes to perform better, we used directed (not one-tailed) statistical tests (Rice & Gaines 1994). This analysis was performed with all introduced fish as well as only with the survivors.

#### (ii) Parasite load, body condition, splenosomatic index and coloration

Surviving fish were tested for significant effects on parasite load with an ANCOVA incorporating body condition at the beginning of the experiment, body condition at the end, sex and enclosure as predictors. We also included all two-way interactions in the model. Thereafter, we correlated the residuals with MHC individual diversity.

As an estimate of the sticklebacks' immunological activation, we calculated a splenosomatic index (SSI) as follows: SSI=(spleen weight/body weight)×100. We performed an ANOVA with SSI as the dependent variable, and enclosure, sex and their interaction as predictors. We then correlated the residuals with individual MHC diversity.

For male breeding coloration, after an ANCOVA was performed to test for the effect of enclosures, body condition at the beginning and at the end of the experiment and of parasite load, we related the residuals of that model to individual MHC diversity.

## 3. Results

### (a) Lifetime reproductive success

**Figure 2:**
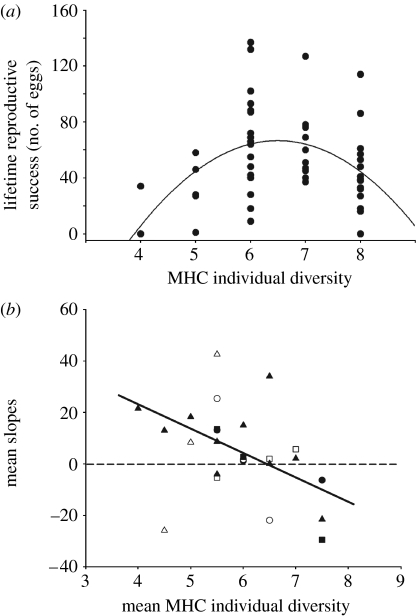
(*a*) Relationship between the LRS (shown as representative number of eggs) and the MHC class II*B* diversity. Fish with an intermediate MHC II B diversity had the highest reproductive output (*n*_eggs_=−0.272+0.136*n*_MHCIIB_−0.499(*n*_MHCIIB_−6.679)^2^, *F*_2,52_=4.29, *p*=0.019). For clarity, raw data of surviving fish are presented but tests were performed on residuals as stated in §2. (*b*) Relationship between the mean slopes of all potential pairs of surviving fish within an enclosure and their relative mean MHC diversity. All data points obtained from the same enclosure are depicted by the same symbol (filled circles, enclosure 1; open circles, enclosure 2; filled squares, enclosure 3; open squares, enclosure 4; filled triangles, enclosure 5; open triangles, enclosure 6). The equation of the linear regression is *f*(*x*)=−27.94*x*+178.81, directed test *F*_1,17_=33.71, *p*=0.0001.

Over the entire breeding season, we collected a total of 149 egg clutches until after seven weeks the nests no longer contained fresh clutches. Using 18 microsatellite loci, 2279 out of the total of 2384 (95.6%) offspring could be unambiguously allocated to a pair of parents. The remaining offspring were removed from subsequent analyses. One per cent of the eggs were evidently stolen from other nests, whereas sneaking behaviour accounted for 12 per cent of the eggs. Hence, although female sticklebacks tend to avoid inbreeding (Frommen & Bakker 2006), but had no choice here, the sneaking rate of males within families in the enclosures was in the same range as in natural populations (Rico *et al.* 1992; Largiadèr *et al.* 2001; Blais *et al.* 2004). The total number of analysed offspring varied between 1 and 137 eggs per individual fish. The LRS of all fish released into the enclosures was significantly correlated only with MHC diversity: fish with an intermediate number of MHC alleles had the highest LRS (figure 2*a*; *n*=96, *t*=−2.40, *p*=0.0185; table 1, *a* and *a1*). None of our other variables could predict LRS (table 1, *a*). This result remained significant when restricted to surviving fish recaptured at the end of the experiment (*n*=53, *t*=−2.55, *p*=0.014; table 1, *a2*) and when recaptured males and females (from 48 of each sex originally introduced) were considered separately (female, *n*=28, *t*=−2.94, *p*=0.007; males, *n*=25, *t*=−2.46, *p*=0.022). Furthermore, LRS was not related to our individual heterozygosity index based on neutral markers (linear: *F*_1,53_=0.155, *p*=0.695; quadratic: *F*_2,53_=0.207, *p*=0.137).

Comparing LRS among sibships: if a genotype with an optimal number of MHC alleles has the highest number of eggs, the slope between egg number of this genotype and that of a genotype having less alleles should be positive, whereas it should be negative between the optimal genotype and a genotype with more alleles. Moreover, two genotypes equally distant from the optimum should have the same egg number, and thus a slope of 0. Furthermore, the mean of the slopes should be positive when the mean of the allele numbers is lower than the optimal number and vice versa. If there is an optimal MHC allele number per individual, all single sibship data points (relationship between the mean slope and the mean MHC variant number of a sibship) should fall on one straight line with negative slope with the *X*-axis intercepting at the hypothesized optimum (see Wegner *et al.* (2003*a*) for a similar analysis and the electronic supplementary material). Indeed, we could detect a significant negative linear relationship between the mean slopes of LRS and the mean number of MHC class II*B* sequence variants (all fish averaged per sibship, directed test *F*_1,5_=5.44, *p*=0.05; surviving fish, directed test *F*_1,5_=7.28, *p*=0.034). This analysis treated each sibship as a statistical unit. With fish averaged for each MHC individual diversity treated as statistical unit, we found similar results (all fish, directed test *F*_1,23_=4.28, *p*=0.031; surviving fish, directed test *F*_1,16_=19.28, *p*=0.0001; figure 2*b*). In the first case, the analysis resulted in an optimum at 6.55 MHC variants, while it resulted in an optimum of 6.40 when considering the surviving fish only. Males had a higher variance in their LRS than females (mean±s.e., males: 55.4±7.92 eggs; females: 53.11±4.57 eggs, Levene test, *F*_1,52_=5.495, *p*=0.023).

### (b) Spleen size

**Figure 3:**
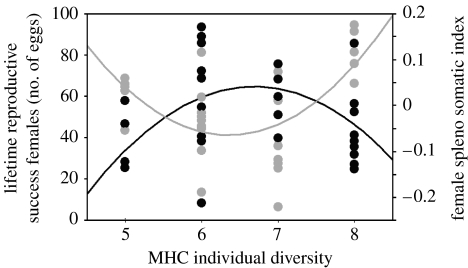
Graph combining the female LRS (shown as representative number of eggs) and SSI as a function of MHC II *B* diversity. Females with an intermediate MHC II *B* diversity having the highest reproductive success are shown by black circles (*n*_eggs_=38.38+4.514*n*_MHCIIB_−11.98(*n*_MHCIIB_−6.481)^2^, *F*_2,26_=4.63, *p*=0.02). Females with an intermediate MHC II *B* diversity having the lowest SSI, representing a better immunocompetence status, are shown by grey circles (SSI=−0.193+0.02*n*_MHCIIB_+0.057(*n*_MHCIIB_−6.536)^2^, *F*_2,26_=6.698, *p*=0.004).

The SSI, an indicator for immune activation, was different among enclosures (*F*_5,51_=7.96, *p*<0.0001), and between the sexes with females showing higher values (*F*_1,51_=6.87, *p*=0.012). Owing to sex differences, we split the dataset and correlated SSI with an individual MHC diversity for each sex separately. Male SSI did not show a significant relationship with MHC diversity (linear, *t*_1,25_=−1.30, *p*=0.206; quadratic, *t*_1,25_=−1.93, *p*=0.083). Females with an intermediate number of MHC variants had the lowest SSI (figure 3; quadratic, *t*_1,28_=3.41, *p*=0.002; linear, *t*_1,28_=1.28, *p*=0.212; table 1, *b*). Moreover, SSI correlated with the total egg number per individual for females (directed test, *t*_1,28_=−1.84, *p*=0.049) but not for males (directed test, *t*_1,25_=0.88, *p*=0.242). Since spleen size is commonly used as a diagnostic tool of the immune system, this result suggests a trade-off between reproduction and immune function only in females.

### (c) Parasite load

We recorded 21 different parasites (for details see the electronic supplementary material) from eight taxonomic groups: Protozoa (*Trichodina* sp., *Apiosoma* sp. and *Ichthyophthirius multifiliis*), Monogenea (*Gyrodactylus* sp.), Digenea (*Diplostomum* sp., *Apatemon cobitidis*, *Phyllodistomum folium*, *Cyathocotyle prussica*, *Tylodelphis clavata*, *Echinochasmus* sp.), Cestoda (*Valipora campylancristrota*, *Proteocephalus filicollis*), Nematoda (*A. crassus*, *C. lacustris*, *Contracaecum* sp., *Raphidascaris acus*), Acanthocephala (*Acanthocephalus lucii* and *Acanthocephalus clavula*), the crustaceans, *Argulus foliaceus* and *Ergasilus* sp., and glochidia, the parasitic larval stages of freshwater mussels (Mollusca).

The total parasite load (table 1, *c*) varied significantly among the enclosures (*F*_5,53_=4.145, *p*=0.004), and females were more infected than males (*F*_1,53_=13.123, *p*<0.001). Infection intensities by trophically transmitted parasites (all nematodes, cestodes and acanthocephalans found in this study) were higher in females than males (*t*_1,52_=3.050, *p*=0.004), whereas there was no difference for directly and actively transmitted parasites (active, *t*_1,52_=1.291, *p*=0.203; direct, Mann–Whitney test, *Z*=0.632, *p*=0.527; see Scharsack *et al.* 2007).

Only for the eye fluke *Diplostomum* sp., but not for the two internal nematode parasites, we could determine the effect of the laboratory infection *in vivo* prior to release into cages. Here, parasite burden increased in the enclosure period significantly (paired *t*-test on log-transformed data, *t*_1,52_=15.994, *p*<0.001) with fish that harboured the highest number of *Diplostomum* sp. at the beginning also showing the highest *Diplostomum* sp. intensity at the end (*R*^2^=0.18, *F*=10.414, *p*=0.002).

### (d) Body condition and coloration

Body condition, a function of a species-specific combination of body length and weight (Frischknecht 1993), was estimated at the beginning and at the end of the experiment for both sexes. The loss in body condition was positively correlated only with parasite burden (*F*_1,52_=9.15, *p*=0.004) but not with MHC individual diversity (linear, *F*_1,52_=0.001, *p*=0.979; quadratic, *F*_1,52_=0.532, *p*=0.469).

Mature male sticklebacks develop distinctive red coloration of the throat, as a secondary sexual character. The intensity of breeding coloration (table 1, *d*) was negatively correlated with parasite load (*F*_1,25_=4.997, *p*=0.0364) but increased with a higher body condition (*F*_1,25_=4.678, *p*=0.0422, see figures in the electronic supplementary material). We did not find any significant differences among families (*F*_5,25_=0.492, *p*=0.778), and the intensity of coloration of males did not predict LRS (*F*_1,25_=0.408, *p*=0.531). Finally, we found no significant correlation between breeding coloration and individual MHC diversity (linear, *F*_1,25_=0.416, *p*=0.526; quadratic, *F*_2,25_=1.00, *p*=0.384).

**Figure 4:**
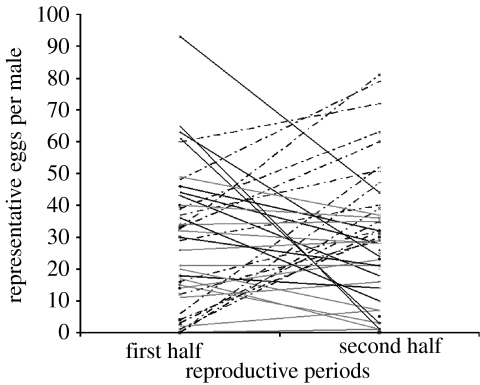
Graph showing the different male reproductive strategies during the experiment. Black dot-dashed lines represent fish that significantly increased the reproductive success in the second half, while black solid lines represent fish that decreased their activity in the second part. Grey solid lines symbolize fish for which there was no difference between the two periods.

Both body condition and coloration of males were measured at the end of the experiment when all fish had ceased reproduction. The missing correlation of either measure with LRS and thus with MHC diversity might result from different males reproducing early and late in the season, as found by Bakker & Mundwiler (1994) in a field study. Also, in the present study, males differed in the time course of their reproductive effort: some reproduced predominantly early and others late (figure 4). The latter had a significantly lower body condition at the end (Welch *t*-test, *t*=−2.151, *p*=0.0384), which suggests that the late reproducing fish were the slightly weaker fish that could reproduce only after the stronger fish had finished, as in Bakker & Mundwiler's (1994) study. However, neither the individual MHC diversity distributions (two-sample Kolmogorov–Smirnov test, *D*=0.1618, *p*=0.9698) nor the LRS (Welch *t*-test, *t*=0.939, *p*=0.3589) differed significantly between the two groups.

## 4. Discussion

Several studies have now identified natural or sexual selection for intermediate rather than maximal MHC diversity (Reusch *et al.* 2001; Aeschlimann *et al.* 2003; Wegner *et al.* 2003*a*,*b*, 2008; Bonneaud *et al.* 2004; Milinski *et al.* 2005; Madsen & Ujvari 2006; Forsberg *et al.* 2007). Here, we show that an intermediate MHC diversity maximizes the LRS, i.e. Darwinian fitness. Our experimental design permitted the estimation of individual LRS because three-spined sticklebacks reproduce only during a single breeding season in their entire life (Wootton 1976). We found that LRS was highest in individuals with an intermediate MHC diversity, which corresponds to an immunogenetic optimum (Wegner *et al.* 2003*a*,*b*; Kurtz *et al.* 2004), even though infected by varied parasite communities in the different enclosures. While this result was significant for both males and females, the mechanistic link of MHC optimality to LRS might differ between the sexes. In our study, body condition and parasite burden are directly linked to LRS probably only in females. Overall, females harboured more parasites than males. However, this does not necessarily indicate lower immunocompetence. The disparity is only due to differential infection intensities of trophically transmitted helminths, thus most likely reflecting different feeding behaviours of females to cover their higher energy demand. The number of eggs a female stickleback can produce is dependent on its physiological capacity and nutritional status (Wootton 1977; Kraak & Bakker 1998). Hence, females need to consume more food (Wootton 1976), which also includes copepods and other potential intermediate hosts. Parasites with active transmission, such as digenean trematodes, did not differ between the sexes in infection intensity.

In males, where the variance in LRS was higher than in females, the situation is more complex, probably because different males had different time courses in their reproductive effort (figure 4), as had been shown by Bakker and Mundwiler (1994) in a field study, possibly indicating a terminal investment strategy of weaker fish. Therefore, neither breeding coloration nor body condition measured at the end of the experiment was correlated with LRS or individual MHC diversity. Because we could not take these measures repeatedly during the course of the experiment, we cannot detect any mechanistic link with individual MHC diversity. However, the reproductive success was measured continuously, allowing us to show that males with an intermediate MHC variant number achieved the highest LRS.

Among condition-dependent male traits in sticklebacks, the intensity of the red breeding coloration is one of the most conspicuous sexual traits that received a lot of attention (McLennan & McPhail 1990; Milinski & Bakker 1990, 1992; Bakker & Milinski 1991; Frischknecht 1993; Bakker & Mundwiler 1994; Candolin 1999; Kraak *et al.* 1999; Barber *et al.* 2000). Parasite infections affect breeding coloration (Milinski & Bakker 1990; Folstad *et al.* 1994) and body condition (Milinski & Bakker 1990; Tierney *et al.* 1996; Blais *et al.* 2004). Accordingly, as shown here, the coloration of males recaptured from the enclosures correlated positively with body condition but negatively with parasite burden measured at the end of the experiment. However, in accordance with previous studies, the quality of sexual ornaments was not greater in males with optimal MHC diversity, but rather could reveal the possession of specific MHC alleles (Buchholz *et al.* 2004; Jäger *et al.* 2007). These respective alleles probably provide resistance against the currently predominating parasite species (Jäger *et al.* 2007). Therefore, female mate choice is predicted to include two criteria: olfactory cues reveal a male's MHC variant diversity, whereas colour reveals the possession of currently protective alleles (Aeschlimann *et al.* 2003; Milinski 2006; Jäger *et al.* 2007). A trait correlating with individual MHC diversity in females was spleen size. Spleen size has been widely used in immunoecological studies as a measure of general activation of the immune system by multiple macroparasite infections, particularly in birds (John 1995; Møller & Erritzoe 1998; Morand & Poulin 2000; Brown & Brown 2002) and fish (Skarstein *et al.* 2001; Kortet *et al.* 2003; Lefebvre *et al.* 2004; Ottova *et al.* 2005). Females with an intermediate number of MHC class II*B* variants had the lowest relative spleen size (figure 4). Spleen size negatively correlated (marginally significant) with the number of eggs assigned to the respective females. This potential link between reproduction, MHC genotype and spleen size is probably due to the activity of the immune system itself, rather than to the result of its efficiency, namely the individual parasite burden. This suggests a trade-off between an individual's LRS and the costs of immunity. Sticklebacks with a more efficient adaptive (MHC dependent) immune system can afford to invest more into their offspring, whereas fish with less optimal MHC diversity need to allocate a higher proportion of their resources for defence mechanisms to maintain their parasite load at tolerable levels. Previous studies have already shown that sticklebacks with an intermediate MHC diversity had the lowest oxidative burst activity, but were nevertheless more capable of limiting the growth of the tapeworm *Schistocephalus solidus* than fish with MHC variant number deviating from this optimum (Kurtz *et al.* 2004). Fish with an optimal MHC diversity basis seem to perform a shift from costly and self-damaging innate immune function towards a probably less costly but efficient adaptive immune strategy, and use the immunological mechanisms more concertedly and economically. Costs of immunity are predominantly regarded as metabolic constraints (Lochmiller & Deerenberg 2000). The results of the present study indicate that immunological costs might directly affect LRS, but that their impact depends to a high degree on the individual MHC genotype. Therefore, these results may further explain why sticklebacks with intermediate MHC diversity prevail in natural stickleback populations (Reusch *et al.* 2001; Wegner *et al.* 2003*a*).

All animal experiments described were approved by the Ministry of Nature, Environment and Country Development, Schleswig-Holstein, Germany.

## Figures and Tables

**Table 1:** Variables correlated with (*a*) LRS, (*b*) SSI, (*c*) coloration and (*d*) parasite load. (Models were chosen on the basis of AIC criteria. ^*^p<0.05, ^**^p<0.001, ^***^p<0.0001.)

terms	estimates	standard error	*t*-ratio	prob>*t*		d.f.	sum of squares	*F*-ratio	prob>*F*
(*a1*) LRS—*polynomial regression*—*optimum*					(*a*) LRS				
MHC class II *B*	0.055	2.907	0.02	0.985	enclosure	5	5156.117	0.992	0.434
MHC class II *B* (quadratic)	−4.378	1.826	−2.40	0.019^*^	sex	1	180.698	0.174	0.679
					parasite load (corrected for enclosure and sex)	1	1189.776	1.142	0.291
(*a2*) LRS surviving fish—*polynomial regression*—*optimum*					initial body condition	1	1175.581	1.129	0.291
MHC class II *B*MHC class II *B* (quadratic)	0.136−0.499	0.2520.195	0.54−2.55	0.5920.014^*^	final body condition (corrected for parasite load and initial body condition)	1	0.006	0.00	0.998
					(*c*) colouration—*ANCOVA*				
					parasite load (corrected for parasite load and body condition)body condition	11	207.987194.716	4.9974.6781	0.0364^*^0.0422^*^
(*b*) SSI—*polynomial regression*—*optimum*									
MHC class II *B*	0.02	0.0157	1.27	0.215	(*d*) parasite load—*ANCOVA*				
MHC class II *B* (quadratic)	0.0567	0.0169	3.36	0.0025^**^	enclosure	5	2.212	4.145	0.0036^**^
					sex	1	1.401	13.123	0.0008^***^
					body condition (corrected body condition and sex)	1	0.086	0.8061	0.3742

## Supplementary Material

Stickleback reproduction in the enclosures–timing and egg numbers

PCR protocols

Prevalence of parasites

How to test for immunogenetic optimum

Male breeding coloration
