# Supplementary material for: Lifetime reproductive success is maximized with optimal major histocompatibility complex diversity
Source: Proc Biol Sci. 2008 Nov 25;276(1658):925–34. doi: 10.1098/rspb.2008.1466 (PMC2664370; doi:10.1098/rspb.2008.1466)
Supplement: Stickleback reproduction in the enclosures–timing and egg numbers [file rspb20081466s48.pdf]

Supplementary Fig. 1: Mean (+/- S.E.) representative number of eggs collected weekly during the experiment. The mean decreased significantly until the last collecting day, indicating that the reproductive period has finished until week 6. Significant differences between the first and last week, and between the last two are depicted by solid horizontal lines.

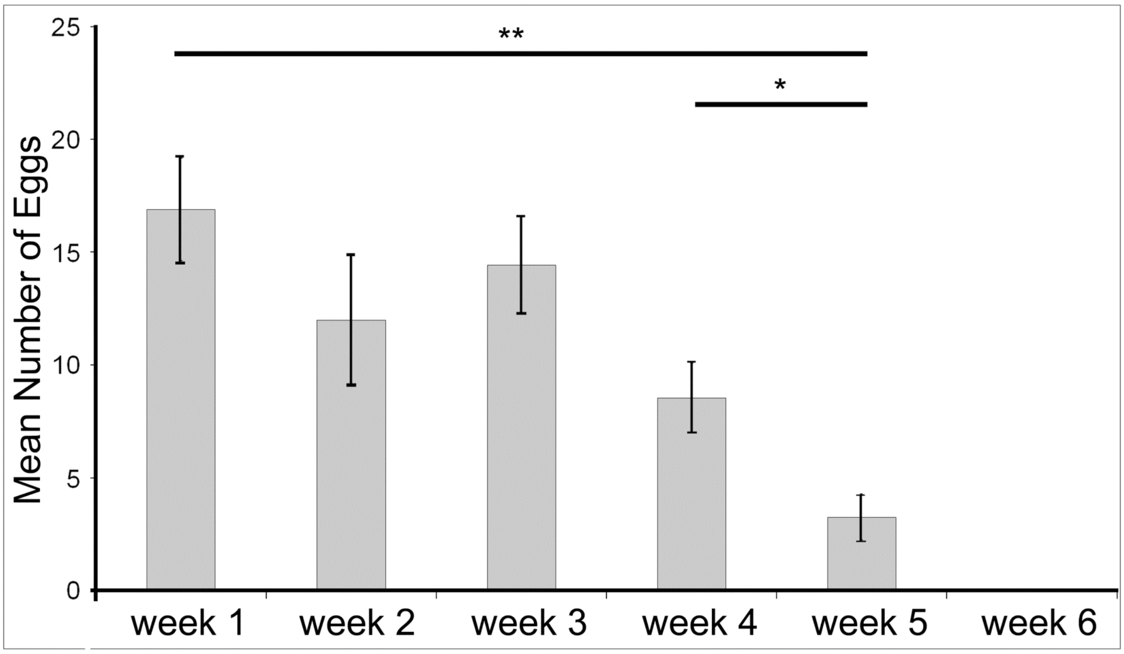

Supplementary Table 1: Number of reproducing sticklebacks recaptured from each enclosure at the end of the experiment (from eight fish per sex and enclosure originally introduced) and mean number of analysed eggs assigned to them.

	N♂ survived and reproduced	N♀ survived and reproduced	Mean N♂ eggs +/- St dev	Mean N♀ eggs +/- St dev
Enclosure 1	7	5	38.4 +/- 26.2	49.6 +/- 16.4
Enclosure 2	5	5	69.8 +/- 47.6	66.8 +/- 24.8
Enclosure 3	6	4	47.6 +/- 44.6	45.0 +/- 11.9
Enclosure 4	2	3	52.5 +/- 19.1	44.3 +/- 12.5
Enclosure 5	2	6	25.0 +/- 9.9	60.0 +/- 17.0
Enclosure 6	3	5	81.3 +/- 44.6	44.8 +/- 34.5

Mean N♂ eggs +/- St dev: Mean number of egg fathered per male +/- standard deviation.
Mean N♀ eggs +/- St dev: Mean number of egg mothered per female +/- standard deviation.
