# Supplementary material for: Lifetime reproductive success is maximized with optimal major histocompatibility complex diversity
Source: Proc Biol Sci. 2008 Nov 25;276(1658):925–34. doi: 10.1098/rspb.2008.1466 (PMC2664370; doi:10.1098/rspb.2008.1466)
Supplement: PCR protocols [file rspb20081466s49.pdf]

Supplementary Table 2: Multiple primer PCR protocols used for microsatellite typing.

PCR 1		PCR2		PCR3		PCR4		PCR5	
10 x buffer	1.00	10 x buffer	1.00	10 x buffer	1.00	10 x buffer	1.00	10 x buffer	1.00
dNTP's	1.00	dNTP's	1.00	dNTP's	1.00	dNTP's	1.00	dNTP's	1.00
% BSA	1.00	1 % BSA	1.00	MgCl2	0.30	MgCl2	0.30	MgCl2	0.30
MgCl2	0.30	MgCl2	0.30	**STN 2 f**	0.30	**STN 36 f**	0.30	**STN 24 f**	0.30
**Stich 5196 f**	0.10	**Stich STN18 f**	0.12	**STN 2 r**	0.30	**STN 36 r**	0.30	**STN 24 r**	0.30
**Stich 5196 r**	0.10	**Stich STN18 r**	0.12	**STN 170 f**	0.30	**STN 114 f**	0.30	**STN 67 f**	0.25
**Stich 4170 f**	0.08	**Stich STN32 f**	0.08	**STN 170 r**	0.30	**STN 114 r**	0.30	**STN 67 r**	0.25
**Stich 4170 r**	0.08	**Stich STN32 r**	0.08	**STN 174 f**	0.30	**STN 167 f**	0.45	**STN 185 f**	0.30
**Stich 1125 f**	0.10	**Stich STN75 f**	0.17	**STN 174 r**	0.30	**STN 167 r**	0.45	**STN 185 r**	0.30
**Stich 1125 r**	0.10	**Stich STN75 r**	0.17	**Taq (Invitek)**	0.05	**Taq (Invitek)**	0.05	**Taq (Invitek)**	0.05
**Stich 1097 f**	0.40	**Stich STN 84 f**	0.08	Water	4.85	Water	4.55	Water	4.95
**Stich 1097 r**	0.40	**Stich STN 84 r**	0.08						
**Stich 7033 f**	0.35	**Taq (Invitek)**	0.05						
**Stich 7033 r**	0.35	Water	4.75						
**Taq (Invitek)**	0.05								
Water	3.59								

All quantities are in µl. The colors represent the label used. Working concentration of the primers: 5 pmol.

Supplementary Table 3: PCR conditions and cycles for each multiple PCR performed

	PCR 1	PCR2	PCR3	PCR4	PCR5
Original Denaturation	3 min at 94°C	3 min at 94°C	2 min at 94°C	2 min at 94°C	2 min at 94°C
**Denaturation**	1 min at 94°C	0.45 min at 94°C	0.45 min at 94°C	0.45 min at 94°C	0.45 min at 94°C
**Annealing**	1 min at 58°C	0.45 min at 56°C	0.45 min at 56°C	0.45 min at 56°C	0.45 min at 56°C
**Extension**	1 min et 72°C	1 min at 72°C	0.45 min at 72°C	0.45 min at 72°C	0.45 min at 72°C
Final extension	None	none	20 min at 72°C	20 min at 72°C	20 min at 72°C
Number of cycle	27	26	27	27	27

In bold the conditions for each cycle
