# Supplementary material for: Lifetime reproductive success is maximized with optimal major histocompatibility complex diversity
Source: Proc Biol Sci. 2008 Nov 25;276(1658):925–34. doi: 10.1098/rspb.2008.1466 (PMC2664370; doi:10.1098/rspb.2008.1466)
Supplement: Prevalence of parasites [file rspb20081466s50.pdf]

Supplementary Table 4: Table summarizing the prevalence (percentage of infected hosts) and the mean intensity (number of parasite individuals of a given species per infected host) of all recorded parasites in the 53 dissected fish.

	**Prevalence (%)**	**Mean Intensity**
Protozoa:		
Trichodina sp.	88.46	18.62
Apiosoma sp.	9.62	5.50
Ichthyophthirius multifiliis	11.54	2.33
Monogenea:		
Gyrodactylus sp.	28.85	17.80
Digenea:		
Diplostomum sp. (total)	100.00	15.98
D. pseudospathaceum (lab infection)	100.00	5.06
Cyathocotyle prussica	98.08	4.35
Echinochasmus sp.	98.08	4.83
Apatemon cobitidis	71.15	4.70
Tylodelphis clavata	98.08	6.93
Phyllodistomum folium	25.00	3.75
Cestoda:		
Valipora campylancristrota	84.62	11.93
Proteocephalus filicolis	86.54	6.87
Nematoda:		
Anguillicola crassus	69.23	1.64
Contracaecum sp.	13.46	1.00
Camallanus lacustris	86.54	4.33
Raphidascaris acus	71.15	2.97
Acanthocephala:		
Acanthocephalus clavula	34.62	2.00
Acanthocepalus lucii	78.85	1.00
Crustacea:		
Argulus foliaceus	15.38	1.91
Ergasilus sp.	9.62	1.00
Mollusca:		
Glochidia	23.08	1.00
