# Supplementary material for: Lifetime reproductive success is maximized with optimal major histocompatibility complex diversity
Source: Proc Biol Sci. 2008 Nov 25;276(1658):925–34. doi: 10.1098/rspb.2008.1466 (PMC2664370; doi:10.1098/rspb.2008.1466)

Testing for immunogenetic optimality by combining several sibships

This is a fictitious example to show how we can test for an optimal number of different MHC class IIB alleles that maximises lifetime reproductive success. The logic of this procedure is the same as in similar analyses for immunogenetic optimality (Wegner et al. 2003a) or optimising MHC heterozygosity for offspring through mate choice (Aeschlimann et al 2003; Milinski 2003). For the present example we combine the data from several sibships each confined in one separate enclosure. Let us assume for simplicity that we find two different genotypes with respect to number of different MHC class IIB alleles in each sibship. Of course more than two genotypes may exist, but the analysis would be similar. Let us assume the two genotypes of sibship one have 2 and 3 different MHC alleles, respectively. Now we determine the mean LRS for each of the two genotypes (supplementary Fig. 2a). If we connect the two values for LRS we find a line with positive slope. So the potential optimum must be higher than 2.5 alleles, which is the average of these two options. In another sibship we find 2 genotypes with 5 and 7 alleles, respectively. Now the slope of the line connecting these two LRS-values is negative (supplementary Fig. 2b, right). This suggests that the potential optimum is lower than 6 alleles, which is the average of these two values. If the average of two options is the optimum number of alleles, the slope of the connecting line should be zero, as in another sibship (supplementary Fig. 2b, middle). We can do this analysis with 6 fictitious sibships. Each sibship gives us a pair of data, namely the average combined allele number and the slope of the line connecting the respective two LRS-values. We can now take each data pair per sibship to a new figure, i.e. the average allele number as x-value and the respective slope as y-value. Now we calculate the linear regression through these 6 slope values (as in Fig. 2b in the main text). The optimum exists if the new regression line has slope, which is significantly different from zero. Then the regression line must have an intercept with the x-axis. This intercept point would we the optimum allele number that maximises LRS. Note that each sibship is a statistical unit providing one pair of data points each. The regression analysis is done with N=6 independent sibships, any pseudoreplication is avoided (Hurlbert 1984; Milinski 1997).

Supplementary Fig. 2: Fictitious example of LRS of two genotypes of each of three sibships. See text for further details.

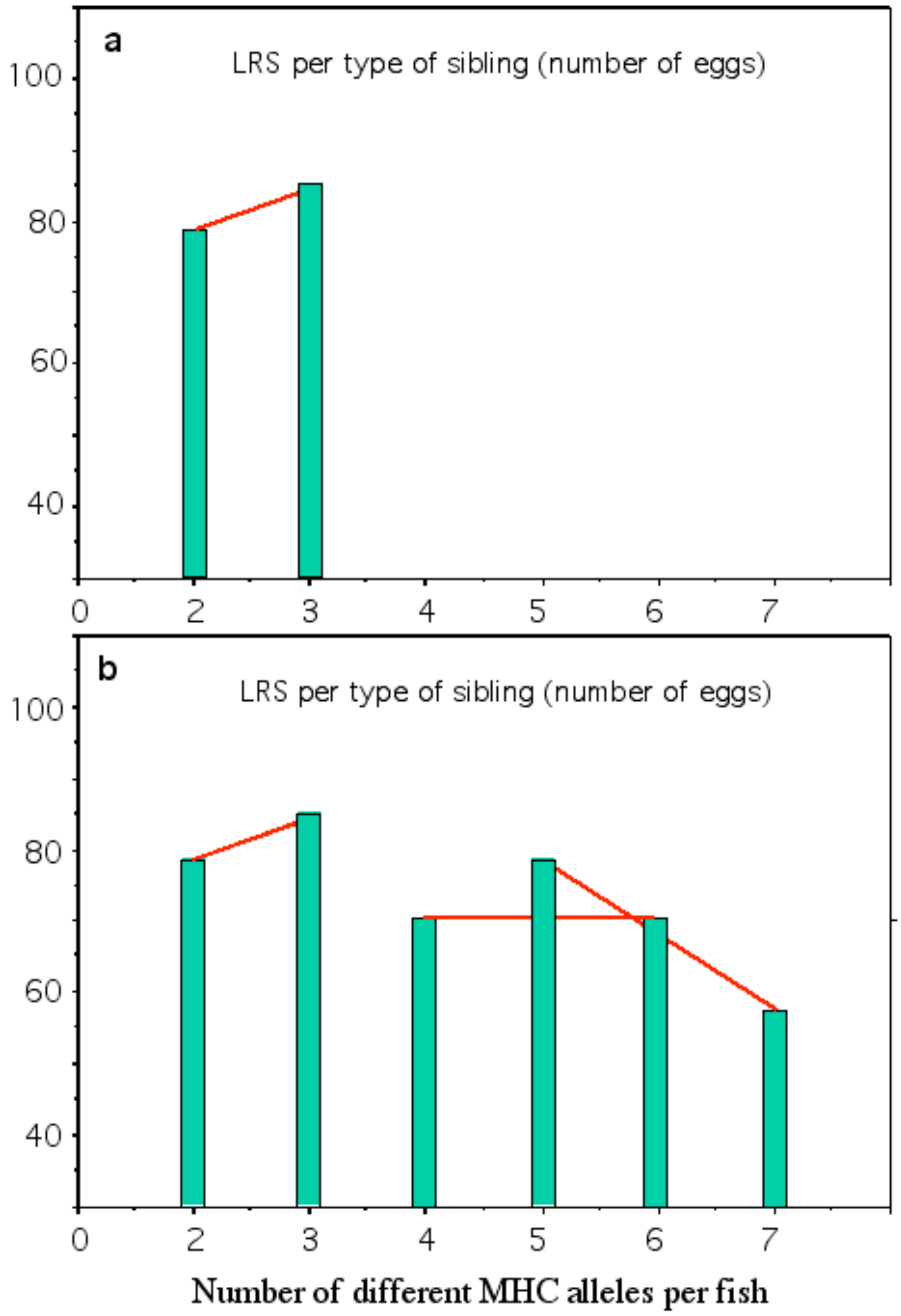

Supplement: How to test for immunogenetic optimum [file rspb20081466s51.pdf]
