# Supplementary material for: Lifetime reproductive success is maximized with optimal major histocompatibility complex diversity
Source: Proc Biol Sci. 2008 Nov 25;276(1658):925–34. doi: 10.1098/rspb.2008.1466 (PMC2664370; doi:10.1098/rspb.2008.1466)

Supplementary Fig. 3: Graph representing the mean intensity of the red colouration of males' throat in relation with individual parasite load. P value of the correlation is indicated on the graph.

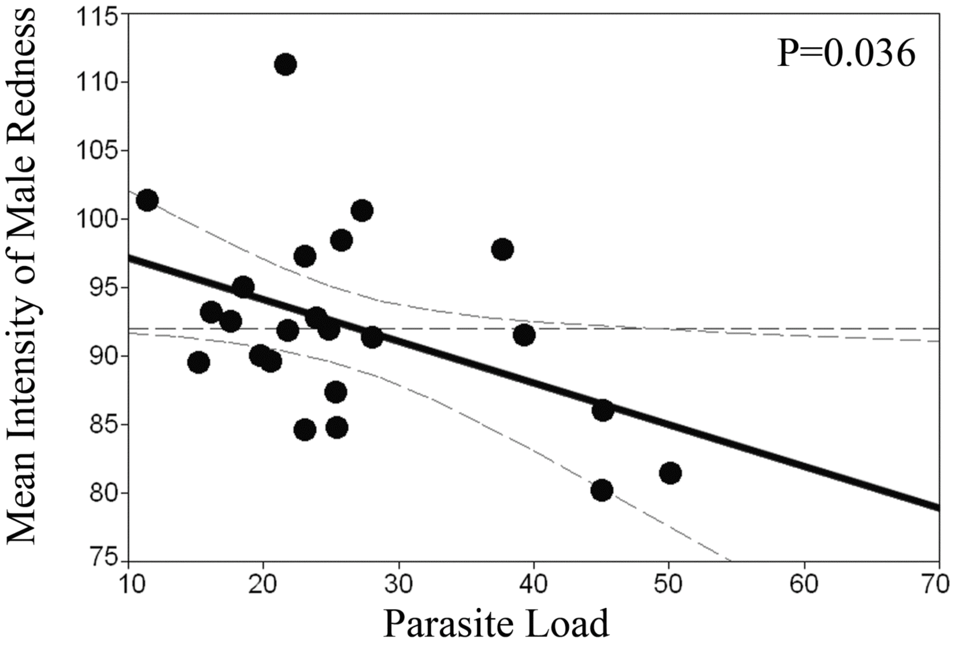

Supplementary Fig. 4: Representation of the correlation between male breeding colouration and standardized final body condition. P value of the correlation is indicated on the graph.

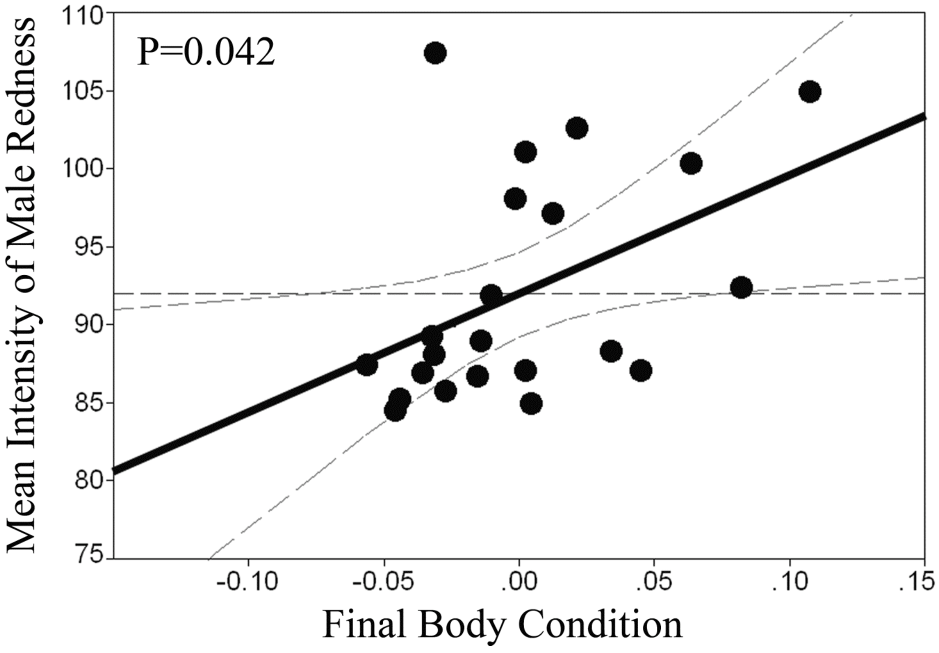

Supplement: Male breeding coloration [file rspb20081466s52.pdf]
